# Uterine rupture in pregnancy after robotic myomectomy

**DOI:** 10.4274/tjod.93609

**Published:** 2015-09-15

**Authors:** İsmet Hortu, Ali Akdemir, Fatih Şendağ, Mehmet Kemal Öztekin

**Affiliations:** 1 Ege University Faculty of Medicine, Department of Obstetrics and Gynecology, İzmir, Turkey

**Keywords:** Uterine rupture, myomectomy, Robotic surgery

## Abstract

Uterine rupture in pregnancy is a rare and often catastrophic complication with a high incidence of fetal and maternal morbidity. A gravida 2 para 1 woman aged 40 years who was 33-34 weeks pregnant presented to our clinic with serious abdominal pain, nausea and vomiting that had begun 6 hours previously. Her past surgical history included a robotic myomectomy 2 years ago in our unit. Obstetric ultrasonography revealed a 33-week fetus without a heartbeat whereupon she underwent emergency laparotomy and we found a 4 cm rupture on the anterior wall of the uterus. Uterine rupture should always be kept in mind, especially in patients with history of uterine surgery.

## INTRODUCTION

Uterine rupture during pregnancy is a rare event and frequently results in life-threatening maternal and fetal compromise. It occurs in 0.5-1% of paptients after laparoscopic myomectomy^(1)^. Uterine rupture occurs when there is a full-thickness disruption of the uterine wall that also involves the overlying visceral peritoneum (uterine serosa). Uterine rupture is divided into two types, complete and incomplete. Incomplete rupture is also known as uterine dehisence in the literature, and comprises tearing of the myometrium and endometrium, but the perimetrium/serosa or visceral peritoneum remains intact. If all uterine layers (endometrium, myometrium, and serosa) tear, this is considered complete rupture. This condition typically occursin the active phase of delivery; however, it can be revealed in pregnancy or after delivery. The initial signs and symptoms of uterine rupture are typically nonspecific, which makes the diagnosis difficult and sometimes delays definitive therapy^(1)^. We report a pregnant woman who underwent emergent surgery in the third trimester of pregnancy for suspected uterine rupture.

## CASE REPORT

A gravida 2 para 1 woman aged 40 years who was 33-34 weeks pregnant was admitted to our hospital with serious abdominal pain, tenderness, nausea and vomitting, which she had had for 6 hours; she reported that there had not been any violence or abdominal trauma.

Our initial physical examination showed that she had a gravid abdomen corresponding to 33-34 weeks of pregnancy and tenderness, with guarding of her lower quadrants of abdomen. Her uterus was tetanic and continuously contracted. The patient’s blood pressure was 100/80 mmHg, pulse 106 beats per minute, SaO_2_: 98%, and her body temperature was 37 ºC.

With the exception of cervical dilation of 1cm, the patient’s other obstetric examination was unremarkable. Obstetric sonography was then performed and a 33-week singleton fetus without heartbeat was found. The placenta was located on the posterior wall of the uterus and no placenta abruptio signs were recorded sonographically, but her Morrison’s pouch and periuterine areas were filled with a collection of free fluid. The patient’s medical history included robotic myomectomy 2 years ago to treat menometrorrhagia and pelvic pain. Her first baby was born with vaginal delivery. Her admission laboratory findings were Hb: 9 gr/dL, Htc: 30%; WBC: 5000/mL; Plt: 350 000/mL. Other laboratory findings were within normal ranges.

**Figure 1:**
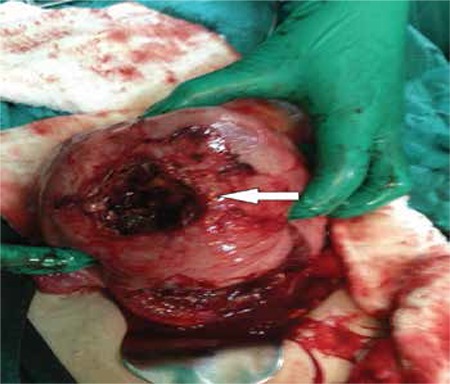
Rupture located on the anterior wall of the uterus (arrow indicates ruptured location)

**Figure 2:**
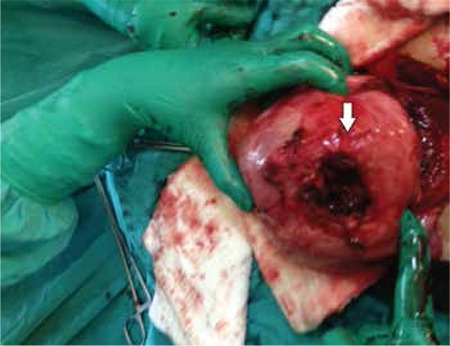
Upper view of rupture

The patient had progressively worsening pain and signs of hypotension. She underwent emergency laparotomy because of the possibility of uterine rupture or placental ablation. During the abdominal exploration, we found that nearly 2 liters free blood had collected in the abdominal cavity and the uterus was contracted; a 4 cm rupture was revealed on the anterior wall of the uterus (Figure 1, 2). The upper abdominal visceral organs (e.g. liver, spleen) and great vessels were normal in appearance. We performed a lower segment cesarean section and a 2990 gr male fetus was stillborn. We repaired the ruptured area with no: 0 Vicryl suture with two layers after cesarean line closure. During the operation her uterus was adenomyotic and contained 3 small millimetric myomas. Her connective tissue, muscles, and especially abdominal wall fascia layers appeared weak. Intraoperatively, we transfused 2 units of erythrocyte suspension. The day after the operation the patient’s physical findings were within normal limits. Her postoperative laboratory findings were Hb: 8 gr/dL; Htc: 26%; WBC: 11 000/mL, Plt: 290 000/mL. The patient was tranfused one more unit of erythrocyte suspension because of the signs orthostatic of hypotension. She was discharged uneventfully three days after the operation.

**Figure 3:**
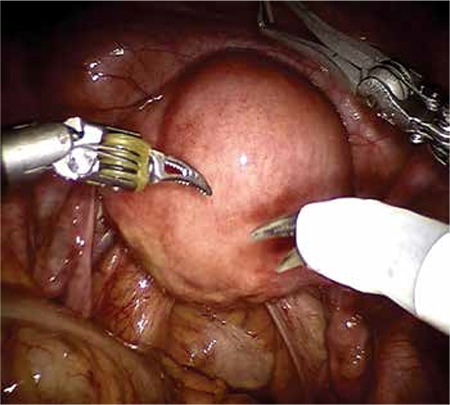
Six cm intramural myoma on the anterior wall of uterus

**Figure 4:**
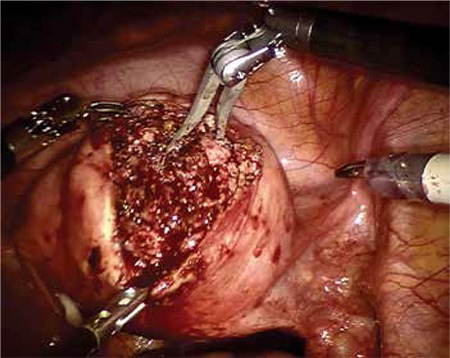
Myoma extirpating using arms

**Figure 5:**
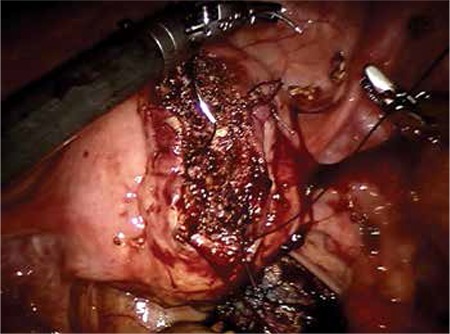
Suturing inner layer of wall defect

**Figure 6:**
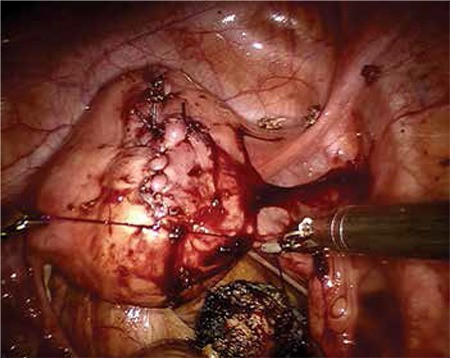
Suturing outer layer of wall defect

**Figure 7:**
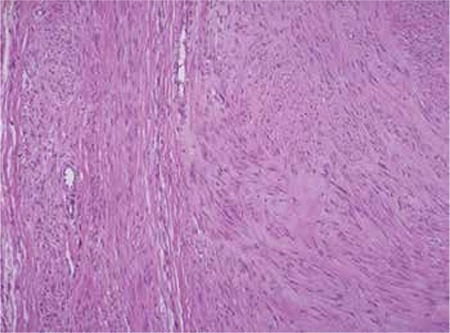
Histopathologic view of excised myoma

Our patient’s surgical history included robotic myomectomy operation 2 years ago to treat menometrorrhagia and pelvic pain. Her robotic myomectomy operation records were reviewed retrospectively. A near 6 cm diameter intramural myoma was extirpated from the anterior wall of the uterus (Figures 3, 4). After myomectomy, the uterine wall defect had been successfully closed using a double-layer no: 0 polydiaxanone suture (PDS) (Figures 5, 6). The myoma’s histopathologic examination was compatible with myoma tissue (Figure 7).

## DISCUSSION

Uterine rupture is a catastrophic tearing of the uterus into the abdominal cavity. Its onset is often only marked by sudden fetal bradycardia and treatment requires rapid surgical attention for better neonatal and maternal outcomes. Predisposing factors include congenital uterine abnormalities, trauma, and other uterine surgical procedures such as myomectomies, and cesarean sections (especially classic vertical). Uterine rupture can occur before or during labor. Today, myomectomy is commonly performed in women with symptomatic fibroids who desire uterine preservation and fertility. The primary surgical techniques used in myomectomy are open surgery, laparoscopic surgery, and recently robot-assisted (robotic) surgery^(2)^. Uterine rupture can either occur in women with a native, unscarred uterus, or in those that have surgical scars from previous surgery^(3)^. Laparoscopic myomectomy was first reported in 1979^(4)^. Laparoscopy has enabled a radical change of practice for surgeons over the last two decades. In general, laparoscopic surgery leads to less scarring, less postoperative pain, and more rapid healing rates compared with laparotomy^(5)^.

Advances in minimally invasive surgery have shaped the field of gynecologic surgery. Robotic-assisted laparoscopy has been proposed as a way to overcome many of the technical challenges in traditional laparoscopy through improved imaging as well as enhanced dexterity of surgical instruments. Robot-assisted laparoscopic surgery has seen rapid progression over the past few years. It has been mostly used for myomectomy, proximal tubal reanastomosis, and deep endometriosis surgery. Technical advantages of robotic-assisted laparoscopic surgery may help overcome these challenges and perhaps improve the adoption of the laparoscopic technique. These advantages include 3-dimensional visualization, instrument articulation, improved dexterity, and the elimination of tremor and counterintuitive movements^(6)^. The advantages of the surgical robot are clearly seen in myomectomy. The wrist motion allows for better, more precise suturing than conventional “straight stick” laparoscopy. The strength of the arms allow for better pulling of the suture, and the third arm for holding the suture under tension^(7)^. Paul et al.^(8)^ reported that uterine rupture during pregnancies following laparoscopic myomectomy was rare following single or multilayer myometrial closure. The authors followed up 217 women subsequent to a laparoscopic myomectomy, 115 of whom had pregnancies. Of the 141 pregnancies, there were 87 cesarean sections, 19 vaginal deliveries, 29 abortions, and 6 ectopic pregnancies. There were no incidents of uterine scar rupture in any of these pregnancies. In another study, Kucera et al.^(9)^ analyzed 69 patients after laparoscopic myomectomy. The authors observed no increase in the incidence of fetomaternal morbidity or severe pregnancy and labor-related complications. There was no uterine rupture after laparoscopic myomectomy in their group. However, other studies have cautioned that every obstetrician should be aware of the morbidity and mortality associated with the critical nature of uterine rupture during pregnancy and/or labor in patients with previous myomectomy^(10)^. Some authors recommend multilayer closure rather than single-layer closure because of previous examples of rupture^(7)^. There is a need for a randomized study to compare single-layer and multilayer suturing techniques. One possible cause of uterine rupture after laparoscopic myomectomy is the wide use of electrosurgery that may result in poor vascularization and tissue (myometrium) necrosis with an adverse effect on scar strength(^11^). Electrocoagulation should be used as sparingly as possible to achieve hemostasis of the edges of after myomectomy. Owing to the risks of electrosurgery, ultrasonic energy (Harmonic scalpel) can be utilized with the robot to perform the hysterotomy compared with bipolar and monopolar energy. In addition to these surgical conditions, the number of myomas, size, location, and types of myoma are other important risk factors for uterine rupture during pregnancy.

Laparoscopic myomectomy should be cautiously performed by a dexterous surgeon. Suture of the wall defect must be carefully performed with multilayer closure in order to avoid uterine rupture during a subsequent pregnancy. Our patient’s operation (uterine wall closure) was undertaken in this way. Also, correct reapproximation of the uterine incision lines depends upon full thickness, evenly-spaced suture placement, thus avoiding hematoma formation. Aggressive electrosurgery used to achieve hemostasis should be avoided^(10)^.

 With this case we realized that healthy and strong connective tissue, and healthy uteruses (no risk factors after uterine surgery, cesarean delivery) have minimal uterine dehissence or uterine rupture risk during pregnancy. However our patient’s abdominal wall’s fascia and muscles were quite weak and her uterine scar was excessively fibromyotic owing to previous surgery. Other potential reasons for uterine rupture include multifetal pregnancy, polyhydramniotic pregnancy, congenital uterine anomalies, and violence or blunt abdominal trauma; our patient had none of these conditions.

In uterine rupture, obstetric clinical symptoms and signs include fetal bradycardia, variable decelerations, fetal death, evidence of maternal hypovolemia, loss of fetal station (detected during cervical examination), and severe or constant abdominal pain. Our patient’s fetus was not alive when she was admitted to our hospital. Diagnosis is confirmed after suspicion through laparotomy; definitive treatment is immediate laparotomy with cesarean delivery and hysterectomy, if necessary. To the best of our knowledge, this case of uterine rupture after robotic myomectomy is the first in the English literature database. Finally, uterine rupture, especially in patients with history of uterine surgery, should always be kept in mind and physicians should be alert to these potentially fatal emergencies.
